# Determining Associations Between Levels of Ethylene Oxide Gas Exposure and Neurocognitive Performance for Older U.S. Adults

**DOI:** 10.3390/ijerph22060852

**Published:** 2025-05-29

**Authors:** Linda O’Kelley, Barbara Swanson, Jessica Bishop-Royse, Joyce W. Tam, Christopher Forsyth, Susan Buchanan

**Affiliations:** 1 College of Nursing, Rush University, Chicago, IL 60612, USA; 2 Department of Psychiatry & Behavioral Sciences, Rush Medical College, Rush University, Chicago, IL 60612, USA; joyce_tam@rush.edu; 3 Department of Internal Medicine, Rush Medical College, Rush University, Chicago, IL 60612, USA; 4 School of Public Health, Environmental & Occupational Health Sciences, University of Illinois Chicago, Chicago, IL 60612, USA; sbucha3@uic.edu

**Keywords:** environmental health, neurocognitive outcomes, ethylene oxide, exposure science

## Abstract

Ethylene oxide (EtO) gas is a widely used industrial chemical and known health hazard. Multiple studies have determined that EtO exposure can be measured via hemoglobin adduct levels, and EtO exposure increases the risk of cancer and neurocognitive deficits, especially with occupational exposure. Emerging studies indicate that neighboring communities are also at risk. The purpose of this study is to explore the relationship of known covariates and EtO hemoglobin adduct levels to neurocognitive performance in older U.S. adults. This exploratory study drew its sample from the publicly available NHANES dataset. The 2013–2014 NHANES measured EtO exposure via hemoglobin adducts and the cognitive domain of neurocognitive function using the CERAD, Animal Fluency, and Digit Symbol Substitution (DSST) tests. Motor function was measured using grip strength. Participants were grouped into background (≤27.36 pmol/gHb) or elevated (>27.36 pmol/gHb) EtO exposure. Hierarchical linear regression, independent *t*-tests, and logistic regression analyses were performed. A total of 10,175 individuals were sampled: 489 were included in the cognitive analyses, and 436 were included in the motor analyss. Elevated EtO adduct levels significantly predicted low Animal Fluency, DSST, CERAD, and combined grip strength scores. Our findings are supported by the extant literature citing neurotoxic EtO exposure effects. Further study in known EtO-exposed communities is warranted.

## 1. Introduction

Since ethylene oxide (EtO) gas was introduced in the 1950s for industrial purposes, it has been linked to serious adverse health outcomes, including cancer and neurotoxic effects [1,2,3,4,5,6,7,8,9,10,11,12,13,14,15,16,17,18,19,20,21,22,23]. Multiple case series and case control studies have found that chronic occupational EtO exposure is associated with impaired cognition, profound forgetting, slower processing speed, sensory loss in the extremities, and muscle weakness [5,6,7,8,13,17,23]. In response, several countries have banned EtO gas use in food production [24].

Most published toxicology studies have focused on exposure in hospital or occupational settings [10,11,12,14,15,16,18]. However, recent data provided by the U.S. Environmental Protection Agency (EPA) indicate the need to look beyond occupational- and hospital-level exposures to also examine chronic, low-intensity exposures, as seen in communities residing near EtO-emitting facilities [19,20]. The few recent studies that have examined the effects of residential EtO exposures report significantly increased risks of hypertension, diabetes, depression, and cancer [3,4,9,22,25]. It remains unknown if the same neurologic symptoms found in occupationally exposed persons are found in persons with residential exposure.

To explicate the association of EtO gas and adverse health outcomes, several studies have identified valid biomarkers of exposure or biological signs that EtO has been absorbed by the body. As EtO gas is rapidly metabolized and has a short half-life [26], EtO biomarkers are more reliable indicators for long-term exposure than EtO blood levels alone [27]. One biomarker of human exposure to toxins is an adduct, a reaction product formed by the conjugation of the toxin with a human macromolecule [28,29]. Most existing EtO biomarkers involve the conjugation of human DNA, hemoglobin, or other proteins with EtO once the gas is metabolized. Researchers have identified biomarkers specific for EtO gas, which include the hemoglobin adduct N-2-hydroxyethylvaline (HEV), the DNA adduct 7-HEG, and the urinary metabolite HEMA [30,31,32].

The EtO hemoglobin adduct is currently being measured in U.S. residents enrolled in the continuous National Health and Nutrition Examination Survey (NHANES) facilitated by the Centers for Disease Control and Prevention (CDC). In the 2013–2014 cycle of the NHANES, the cognitive and motor domains of neurocognitive function were also concurrently measured for a subsample of participants and included the Consortium to Establish a Registry for Alzheimer’s Disease (CERAD) Word Learning subtest, Digit Symbol Substitution test, Animal Fluency test, and grip strength test. These tests measure cognitive and motor domains known to be affected by environmental neurotoxins, such as lead, cadmium, manganese, mercury, and arsenic [33,34,35,36,37,38,39]. Multiple studies have found these tests to be sensitive for detecting the cognitive and/or motor effects of environmental contaminants in large, exposed populations [33,34,35,36,37,38,39]; however, to our knowledge, there have been no studies examining the link between levels of EtO hemoglobin adducts and cognitive/motor function in exposed populations.

Thus, the purpose of this study is to explore the relationship of EtO hemoglobin adduct levels, known predictors of neurocognitive function, and smoking status to cognitive and motor performance in a sample of older U.S. adults using the NHANES database. As there is limited research on the effects of EtO gas at the community level, this novel study will expand the current understanding of the risk posed by community-level EtO gas exposure.

## 2. Materials and Methods

Study Design

This project is a secondary analysis of the 2013–2014 NHANES dataset, conducted by the National Center for Health Statistics (NCHS), a division of the CDC. The NHANES study is a large, continuous, and publicly available dataset that documents statistics for a wide variety of health- and nutrition-related measures for a sample of approximately 10,000 participants per two-year cycle.

Sample and Setting

Using purposive sampling, NHANES recruits a sample that is representative of the U.S. population in terms of age, biological sex, race and ethnicity, and income within each cycle [40,41]. NHANES chooses 30 counties per two-year cycle and randomly selected neighborhoods and households within the chosen counties using U.S. Census Bureau information [42]. If consenting participants meet the eligibility requirements, they undergo an interview and physical examination, including the collection of blood and urine specimens [40]. The CDC anonymized this dataset and removed protected health information to ensure confidentiality for study participants, including zip codes and/or other location data [43,44].

The 2013–2014 cycle is the only known cycle published to date that includes neurocognitive and EtO hemoglobin adduct data. The neurocognitive battery includes (a) three cognitive tests administered to participants aged 60 years and older and (b) a grip strength motor test administered to participants 6 years of age and older. Of the approximately 10,000 NHANES participants aged 6 years and older, the EtO hemoglobin adduct was quantified in a randomly selected subsample of 1/3 of the participants (N ≈ 300) [45].

*Inclusion and Exclusion Criteria*

The inclusion criteria for the NHANES dataset included noninstitutionalized, civilian U.S. residents [40]. The exclusion criteria consisted of the following: (a) non-U.S. citizens, (b) U.S. citizens residing outside of the U.S., (c) all active-duty military personnel, (d) hospitalized persons, and (e) incarcerated persons [40]. In this study, only those participants who had values for EtO hemoglobin adduct levels *and* who completed all the cognitive and motor tests were included in the final analyses. Cognitive function measures were only administered to participants aged 60 and over. Accordingly, our sample consisted of persons ≥ 60 years of age, and we excluded grip strength data collected from participants younger than age 60.

Measures
*Demographic Variables and Smoking Status*

Demographic data and biomarkers of cigarette smoke exposure were collected via interviews or blood draws as part of the NHANES study. The collected demographics included age, biological sex, income, education level, race/ethnicity, and language spoken. The reference categories for the categorical variables included the following: biological sex: female; education level: college graduate; race/ethnicity: non-Hispanic White; and language spoken: English. The sample in the current study was limited to participants aged 60 and over. Participants aged 80 and above were top coded into the 80+ group per NHANES procedure. Cigarette smoke exposure was included in the analyses because smokers have higher levels of EtO biomarkers in their blood [46]. Serum cotinine levels were used to determine cigarette smoke exposure for both active smokers and those exposed to secondhand smoke, with higher levels indicating higher levels of exposure [47]. Serum cotinine levels are widely used, over nicotine alone, as a biomarker of cigarette smoke exposure due to cotinine’s longer half-life and, therefore, more accurate indication of long-term cigarette smoke exposure [47].

*Cognitive and Grip Strength Measures*

The three cognitive assessments administered during the 2013–2014 cycle of NHANES were included in the analyses: the CERAD Word List Learning test (CERAD W-L), Animal Fluency test, and Digit Symbol Substitution test (DSST). These assessments are commonly used to evaluate cognitive function, including memory (CERAD W-L), semantic fluency (Animal Fluency), and processing speed (DSST) [48,49,50]. The CERAD W-L measure contains 3 immediate learning trials and a delayed recall trial, which yield a total learning score (i.e., the sum of the raw scores across the three learning trials) and a delayed recall score. For all three cognitive assessments, higher scores indicate better cognitive performance. All the cognitive tests were administered privately in mobile examination centers by trained interviewers [51].

Under the umbrella of neurocognitive function, both cognitive and motor domains were assessed within the 2013–2014 cycle of NHANES. Similar to cognitive function, motor function is related to brain processing, with some studies indicating that motor dysfunction can precede cognitive dysfunction in the context of dementia [52]. Grip strength, which measures the muscle strength of the hands, represents the motor domain of neurocognitive function. Combined grip strength was calculated as the sum of the largest dynamometer reading from three trials in each hand and measured in kilograms (kg) [53].

*Ethylene Oxide Blood Levels*

The gold standard for quantifying EtO exposure is the hemoglobin adduct, HEV, level in human whole blood [1,30,32,42]. To quantify HEV levels, peripheral venous blood specimens were collected from randomly selected, eligible NHANES participants in mobile examination centers. To ensure consistency among the collection sites, all specimens were handled following a detailed protocol concerning specimen collection, processing, storage, and shipping [42,44]. At the CDC laboratory, a modified Edman reaction was performed to isolate the EtO hemoglobin adduct, followed by high-performance liquid chromatography and tandem mass spectrometry. Analyses were primarily conducted in singlets; however, approximately 2% of the sample was randomly selected to be analyzed in duplicate for a quality analysis. EtO hemoglobin adduct levels are expressed as pmol/g Hb.

The participants were categorized into two groups based on their EtO hemoglobin adduct concentrations: background (≤27.36 pmol/g Hb) or elevated (>27.36 pmol/g Hb). This conversion from a continuous-level to a binary variable was necessary as the highly positive skew violated assumptions for the planned analyses. Log and square root transformation were attempted but unsuccessful. As there are no defined background or normal level cut-offs for EtO hemoglobin adduct levels, these categories were created based on the positively skewed distribution of the EtO hemoglobin adduct data observed in our sample. The cutoff of 27.36 pmol/g Hb was selected as 73.6% of the participants showed hemoglobin adduct levels between the narrow range of 5–27.36 pmol/g Hb. A wider distribution from 27.37 pmol/g Hb to 989.35 pmol/g Hb represents the participants with elevated levels.

Procedures

Rush University IRB approval was obtained. Data from the 2013–2014 NHANES cycle were accessed and downloaded from the publicly available website and imported into SPSS.

Data Analysis

All statistical analyses were conducted using IBM SPSS (v. 30), using sample weights, as per NHANES guidelines. To preserve an appropriate sample size given the large number of missing variables in the NHANES database, missing data were excluded pairwise. This approach allowed cases with missing data on variables that were irrelevant to the study purpose to remain in the analysis when variables of interest had non-missing values.

The analysis was conducted using corrected laboratory values of EtO hemoglobin adduct levels that were released by the CDC in May of 2023. This correction was necessary due to a discovered calibration error in commercially purchased equipment at the CDC laboratory that led to a systematic bias in original results. The data were corrected via log transformation using the following formula: *LBXEOA* = 0.58026 (*LBXEOA_original* ^1.03562^). The original results were withdrawn from the NHANES website in March 2022 and were not available for analysis [45].

*Variable Selection Analysis*

To evaluate the impact of EtO exposure on cognitive and motor performance, variable selection analyses were undertaken to determine which demographic characteristics significantly related to scores on the neurocognitive measures and to other potential predictors. A larger sample size of 1392 to 1573 participants in the 2013–2014 NHANES (without the inclusion criterion of published EtO hemoglobin adduct levels) was used to verify covariates to increase the power of the analyses. Multivariable linear regression models examined associations between the available predictors and performance on each of the cognitive (CERAD-WL learning trials, CERAD delayed recall, Animal Fluency, and Digit Symbol Substitution) and motor (combined grip strength) tests.

*Hierarchical Linear Regression*

To explore the relationship between the binary EtO hemoglobin adduct levels and neurocognitive function, while controlling for the identified covariates, a Pearson correlation coefficient and *R*^2^ value were calculated using hierarchical linear regression models for each neurocognitive measure. Variables were added in blocks, with relevant covariates, including age, income, smoking status, gender, education level, race/ethnicity, and language spoken, being included in the first step of the model. Relevant covariates and EtO adduct levels were included in the second step. This process allowed for the relationship between EtO levels and neurocognitive function to be explored, while controlling for the effects of relevant demographics.

The assumptions of regression models were evaluated prior to analysis [54]. We found little evidence for violations of these assumptions, with the exception of outliers and high leverage points observed in some models. Outliers were subsequently removed to satisfy and facilitate the analysis. Observations representing high leverage points were retained as their removal would have resulted in participants with the “other” race category being dropped from the analysis.

*Independent samples t-test*

To compare the cognitive function of persons with background and elevated EtO hemoglobin adduct levels, an independent samples *t*-test was performed. A *t*-test statistic and a significance value were calculated to determine if significant group differences were present for background vs. elevated EtO adduct levels for each of the 5 neurocognitive assessments.

All relevant assumptions were tested prior to analysis and met, with the exception of outliers in each model. Outliers were subsequently removed to satisfy assumptions and facilitate the analysis.

*Logistic regression analysis*

To compare the odds of having low cognitive scores for background vs. elevated EtO hemoglobin adduct levels, an odds ratio, confidence interval, and significance value were calculated using a hierarchical logistic regression for each cognitive test. All cognitive data were adjusted by age based on manual or published normative data [55,56,57]. For the CERAD test, performance was further adjusted by education level (less than a high school degree or a high school degree and beyond) [57]. Z-scores were calculated to identify cognitive impairment, using a binary variable (present/absent). Impaired cognitive function was defined as scoring 1.5 SD below the normative mean, a conventional criterion for impaired cognitive performance [58,59,60]. All the relevant assumptions were tested prior to analysis and met.

## 3. Results

The number of participants who met the eligibility criteria and completed each of the neurocognitive tests ranged from 436 (motor) to 489 (cognitive) (Table 1). The mean age of the participants was 68.98 years, and they were equally distributed by biological sex. There were notably larger groups of non-Hispanic White individuals, English speakers, and those with some college or above education among those NHANES participants who completed the five neurocognitive assessments (Table 1). The mean EtO hemoglobin adduct level was 37.17 pmol/g Hb, with 333 of the participants classified as having background levels and 156 of the participants classified as having elevated levels. We found that the NHANES participants with elevated EtO hemoglobin adduct levels were significantly younger, with higher levels of cigarette smoke exposure, lower income, more diverse, and had lower education levels compared to the participants with background levels (Table 2). The participants’ performance on each of the neurocognitive outcome measures is presented in Table 3.

### 3.1. Variable Selection Analyses

Age, income, biological sex, race/ethnicity, education, and language spoken were significantly associated with each cognitive measure and were, therefore, included as covariates in the final analyses for cognitive performance. Except for language spoken, the same factors were significantly correlated with grip strength and were included as covariates. While smoking status did not correlate with neurocognitive performance, it was included in the final analyses as a covariate due to the known association between higher serum cotinine levels and higher levels of EtO adducts in the blood [46].

### 3.2. Relationship Between EtO Levels and Neurocognitive Performance

Multiple hierarchical linear regression analyses were performed to investigate if elevated EtO hemoglobin adduct levels were significantly related to neurocognitive performance when controlling for available covariates. The full model of neurocognitive performance (age, smoking status, income, biological sex, race/ethnicity, education, language spoken) and elevated EtO hemoglobin adduct levels were calculated for each of the five neurocognitive measures. For the CERAD delay recall, the participants who were older; of the male biological sex; of Hispanic, Black, or other race descent; and had elevated EtO hemoglobin adduct levels performed more poorly (Table 4, Model 2b). Elevated EtO hemoglobin adduct levels accounted for approximately 2% of the variation in scores, *F*(1,15) = 9.31, *p =* 0.008. A significant inverse relationship between elevated EtO hemoglobin adduct levels and motor performance, as measured by combined grip strength, was also found in the full model (Table 5). Elevated EtO hemoglobin adduct levels accounted for less than 1% of the variation in scores, *F*(1,15) = 8.84, *p* = 0.009. Models for the CERAD-WL learning trials, Animal Fluency test, and DSST were non-significant (Table 4, Models 1, 3, & 4).

### 3.3. Group Differences Between Background and Elevated EtO Adduct Levels

Independent *t*-tests were performed to determine if significant differences existed between the background and elevated EtO groups’ neurocognitive mean scores. The participants with elevated EtO hemoglobin adduct levels performed significantly worse than those with background levels on the CERAD delayed recall, *t*(15) = 3.53, *p* = 0.003, Animal Fluency, *t*(15) = 2.67, *p* = 0.018, and Digit Symbol Substitution tests, *t*(15) = 2.24, *p* = 0.041. No significant group differences were found for the CERAD-WL learning trial sum or the combined grip strength test (Table 6).

### 3.4. Predictors of Low Cognitive Scores

A logistic regression analysis was performed to assess the association between elevated EtO hemoglobin adduct levels and the likelihood of obtaining a low score on the neurocognitive tests. After adjusting for age and education, elevated EtO hemoglobin adduct levels significantly predicted low cognitive scores (defined as scoring ≥ 1.5 SD below the normative mean) for the CERAD-WL trial #1, χ^2^(1) = 7.95, *p* = 0.005, and CERAD delayed recall test, χ^2^(1) = 4.93, p = 0.026, as compared to background levels. Elevated EtO hemoglobin adduct levels were found to increase the odds of scoring low on the CERAD-WL trial #1 by 2.09× and on the CERAD delayed recall test by 1.76× (Table 7). After adjusting for age, elevated EtO hemoglobin adduct levels significantly predicted low cognitive scores for the Animal Fluency test, χ^2^(1) = 9.98, *p* = 0.002, increasing the odds of scoring low on the Animal Fluency test by 1.79× (Table 7). As the available normative data for the Animal Fluency test accounted for age only, additional analyses were performed to account for education. After accounting for age and education (< or ≥12 years of education), the Animal Fluency test was not found to be significant. Models for the CERAD-WL trial 3 and the DSST were not significant (Table 7).

We completed additional analyses to understand the demographics of the participants in our sample who scored in the impaired cognitive function range. We compared the age, education level, and race/ethnicities of the participants who received a higher number of impaired cognitive scores (3–5) to those who received no or few impaired cognitive scores (0–2). A χ^2^ analysis was performed with all assumptions met. We found significant associations between age (*p* = 0.014) and race/ethnicity (*p* = 0.007) and the number of low cognitive scores received, indicating that those who were older and with diverse race/ethnicities had more scores in the impaired cognitive function range. Education differences were non-significant.

## 4. Discussion

To our knowledge, this study was the first to evaluate associations between EtO gas exposure and cognitive and motor performance in a representative population of older U.S. adults. We found significant inverse associations between elevated EtO hemoglobin adduct levels and neurocognitive performance on the CERAD-WL learning trial, CERAD delayed recall, Animal Fluency, DSST, and combined grip strength tests. Our results suggest that elevated levels of EtO hemoglobin adducts are associated with poorer performance in memory, semantic fluency, processing speed, and muscle strength.

We also found that the participants with elevated EtO hemoglobin adduct levels had significantly increased odds of low cognitive scores when compared to normative samples, adjusting for age and education when possible. This is significant as those participants with elevated EtO hemoglobin adduct levels not only scored lower than those with background levels, but their scores were also lower compared to cognitively healthy samples of individuals.

It was hypothesized that the participants with elevated EtO hemoglobin adduct levels were more likely to have low cognitive scores due to the combination of (a) the neurotoxic effects of EtO gas exposure and (b) the higher representation of socially and economically disadvantaged groups who generally face a higher risk of co-occurring environmental hazards [61,62]. In support of the latter, we found significant differences in race/ethnicities between the background and elevated EtO hemoglobin adduct groups. These findings highlight the need for further studies of residential EtO gas exposure to address both the significant neurocognitive deficits and social justice concerns related to exposure.

The current findings were consistent with the existing literature indicating the neurotoxic effects of EtO exposure. Over several decades of investigation, multiple researchers have published case reports and case series documenting cognitive dysfunction and physical symptoms in occupationally exposed persons [5,6,7,8,13,17,23,63,64,65,66,67,68,69]. Reported symptoms range from headaches and neuropathy to memory loss and poorer psychomotor speed [5,63,70]. These reported effects comprise both cognitive and motor functioning, suggesting an association between elevated EtO adduct levels and dysfunction across multiple neurocognitive domains. Because our study only analyzed four cognitive measures, we were not able to replicate these findings in our sample of community dwelling persons with EtO exposure from unknown sources. 

While statistically significant cognitive and motor differences were found between the participants with elevated vs. background EtO hemoglobin adduct levels, the associations were relatively weak, with small effect sizes after accounting for covariates. Combined with the absence of measures of functional outcomes, further inferences regarding clinical relevance cannot be made. The neurocognitive dysfunction described in the extant literature is related to occupational exposure, where EtO gas levels are reported to be orders of magnitude higher than found with residential exposure to the ambient air. Additionally, persons with a lower SES are more likely to be exposed to high levels of multiple co-occurring environmental hazards and chemicals, possibly confounding the explication of effects specific to EtO gas exposure [61,62]. Nevertheless, this exploratory analysis is an important first step to better understand the relationship between EtO gas exposure and neurocognitive sequelae.

Further examination of community-dwelling populations in known areas of EtO exposure is warranted. More targeted studies in a wider age range are recommended as the literature suggests that children and pregnant women are highly susceptible to the effects of environmental exposures, particularly air toxins [71]. In addition to the CERAD, Animal Fluency, DSST, and combined grip strength tests, a more comprehensive test battery should be considered. Recommended tests include those that measure neurocognitive domains of visual memory, visuospatial skills, other aspects of language (e.g., phonemic fluency and naming), and executive functioning, as these domains were not assessed in the 2013–2014 cycle of NHANES.

### Limitations

While this study may be the first to examine neurocognitive EtO exposure effects in the U.S. population, multiple limitations arise from working exclusively with NHANES data. First, the study sample was limited to noninstitutionalized, older U.S. adults, and the results cannot be generalized to the general U.S. population. Second, we were unable to determine if the participants had occupational exposure to EtO gas. Additionally, it is unknown if the participants sampled from the 2013–2014 cycle of NHANES resided near an EtO-emitting facility. As NHANES demographic data are restricted, we were unable to access geographic data at the zip code level to determine the EtO exposure source. Third, NHANES participants represent a sample with an unknown cognitive status. While NHANES restricts eligibility to noninstitutionalized persons, some participants completing the cognitive tests may have had some degree of existing cognitive impairment.

Additionally, there are analytical limitations that should be noted. First, normative data for each neurocognitive measure were not co-normed. While all three neurocognitive normative test scores accounted for age, only the CERAD scores also accounted for education and biological sex. Additionally, the normative scores were typically several decades old and likely calculated from homogeneous populations, thus raising questions about how accurately they represent the true normative scores in the U.S. population [72]. Second, no normative scores were found by the authors for grip strength, excluding this test from the normative analyses. Third, while most assumptions were met or corrected by removing outliers, cases with high leverage points remained in the final hierarchical linear regression analyses to retain individuals identifying as other races in the analytical model.

## 5. Conclusions

We found significant inverse associations between EtO hemoglobin adduct levels and both cognitive and motor domains of neurocognitive performance in older U.S. adults. In some cases, these findings are above and beyond the significant influences of known covariates including age, race, income, biological sex, smoking status, education level, and language spoken. These findings are supported by a large body of extant literature on the neurotoxic effects of EtO gas exposure, but further research is still needed. Additional studies using a sample representative of the U.S. population in terms of age and in known areas of EtO gas exposure are recommended.

## Figures and Tables

**Table 1:** Weighted descriptive statistics of predictors: NHANES demographics and serum cotinine levels for participants completing cognitive (N = 489) and motor (N = 436) tests.

Characteristic	N	%	M	SD
HbEtO Adduct Levels			37.17	3.04
Background Levels	333	73.6		
Elevated Levels	156	26.4		
Age (Measured in Years) (N = 489)			68.98	0.50
Serum Cotinine Levels (N = 480)			37.05	6.03
Ratio of Family Income/Poverty (N = 456)			3.05	0.11
Gender (N = 489)				
Men	238	49.0		
Women	251	51.0		
Race/Ethnicity (N = 489)				
Hispanic	91	7.0		
Non-Hispanic White	256	81.0		
Non-Hispanic Black	92	7.8		
Non-Hispanic Asian	46	3.8		
Other Race (including multi-racial)	4	0.4		
Education (N = 489)				
Less Than 12th Grade	112	13.1		
Highschool Grade or Equivalent	123	23.2		
Some college or AA degree	136	31.1		
College Graduate or Above	118	32.6		
Language for Cognitive Tests (N = 489)				
English	429	95.1		
Spanish	48	3.9		
Asian Languages	12	1.0		
Language Used for Grip Strength (N = 436)				
English	405	97.6		
Spanish	31	2.4		

**Table 2:** Weighted demographics of sample, comparing participants with background (N = 333) and elevated (N = 156) EtO hemoglobin adduct levels.

	Background		Elevated		Group Differences	
	M	SD	M	SD	*F*	*p*
Age (Measured in Years) (N = 489)	69.02	0.58	68.88	0.77	5.71	0.017
Serum Cotinine levels (N = 480)	8.14	5.75	117.21	27.47	87.36	<0.001
Ratio of Family Income/Poverty (N = 456)	3.18	0.13	2.70	0.15	9.31	0.002
	N	%	N	%	*χ* ^2^	*p*
Biological Sex (N = 489)					2.82	0.073
Men	148	46.8	90	55.4		
Women	185	53.2	66	44.6		
Race/Ethnicity (N = 489)					12.30	<0.001
Hispanic	63	6.6	28	8.0		
Non-Hispanic White	191	84.3	65	71.9		
Non-Hispanic Black	52	6.0	40	12.6		
Non-Hispanic Asian	25	2.7	21	7.0		
Other Race (including multi-racial)	2	0.3	2	0.6		
Education (N = 489)					15.23	0.030
Less Than 12th Grade	71	12.6	41	14.7		
Highschool or Equivalent	76	19.9	47	32.5		
Some College	102	34.1	34	22.5		
College Graduate or Above	84	33.4	34	30.3		
Language for Cognitive Tests (N = 489)					2.86	0.018
English	299	95.8	130	92.9		
Spanish	29	3.6	19	5.0		
Asian Languages	5	0.6	7	2.2		
Language Used for Grip Strength (N = 436)					0.23	0.841
English	279	97.7	126	97.4		
Spanish	22	2.3	9	2.6		

**Table 3:** Descriptive statistics of NHANES scores for cognitive (N = 489) and motor function tests (N = 436).

Outcome Variable	M	SD
CERAD List Learning Total	20.56	0.29
CERAD Delayed Score	6.54	0.16
Animal Fluency Test Score	18.06	0.33
Digit Symbol Substitution Test Score	51.24	1.05
Combined Grip Strength Score	64.75	1.42

**Table 4:** Hierarchical linear regression of available predictors of cognitive performance and elevated HbEtO adduct levels on cognitive function.

Model	1a	1b	2a	2b	3a	3b	4a	4b
	CERAD-WL Learning Total		CERAD Delay Recall		Animal Fluency		Digit Symbol Substitution Test	
	Step 1	Step 2	Step 1	Step 2	Step 1	Step 2	Step 1	Step 2
Age (Years)	−0.22 (0.03) ***	−0.22 (0.03) ***	−0.12 (0.02) ***	−0.12 (0.02) ***	−0.29 (0.05) ***	−0.29 (0.04) ***	−0.81 (0.07) ***	−0.81 (0.07) ***
Serum Cotinine	0.00 (0.00)	0.00 (0.00)	0.00 (0.00)	0.00 (0.00)	−0.00 (0.00)	−0.00 (0.00)	−0.01 (0.00) **	−0.01 (0.01) *
Income/Poverty Ratio	0.40 (0.12) **	0.39 (0.12) **	0.18 (0.08) *	0.16 (0.07) *	0.28 (0.23)	0.25 (0.24)	3.60 (0.40) ***	3.60 (0.40) ***
Male Gender (ref = female)	−1.98 (0.32) ***	−1.93 (0.30) ***	−0.84 (0.11) ***	−0.79 (0.11) ***	−0.46 (0.57)	−0.38 (0.60)	−6.78 (1.64) ***	−6.79 (1.71) **
Race/Ethnicity (ref = white)								
Hispanic	−0.82 (0.56)	−0.77 (0.53)	−0.69 (0.26) *	−0.62 (0.26) *	−0.52 (1.04)	−0.43 (1.07)	−7.48 (2.35) **	−7.49 (2.36) **
Black	−1.02 (0.64)	−0.94 (0.63)	−0.66 (0.21) **	−0.56 (0.21) *	−3.04 (0.80) **	−2.90 (0.79) **	−8.75 (1.11) ***	−8.76 (1.10) ***
Asian	−1.43 (0.82)	−1.28 (0.82)	−0.63 (0.40)	−0.44 (0.40)	−3.43 (0.60) ***	−3.17 (0.71) ***	−3.86 (2.79)	−3.88 (3.04)
Other	−4.43 (1.62) *	−4.39 (1.63) *	−1.53 (0.49) **	−1.48 (0.53) *	−1.69 (2.46)	−1.62 (2.39)	−5.63 (2.00) *	−5.64 (1.95) *
Education (ref = college grad)								
<12th Grade	−1.26 (1.02)	−1.35 (0.98)	−0.16 (0.52)	−0.27 (0.53)	−3.80 (1.09) **	−3.95 (1.07) **	−7.48 (3.44) *	−7.46 (3.42) *
Highschool	−0.82 (0.60)	−0.77 (0.61)	−0.50 (0.38)	−0.44 (0.37)	−3.93 (0.81) ***	−3.84 (0.80) ***	−3.62 (2.81)	−3.63 (2.90)
Some College	−0.17 (0.68)	−0.26 (0.65)	−0.06 (0.41)	−0.17 (0.39)	−1.78 (1.05)	−1.93 (1.03)	0.75 (2.81)	0.76 (2.60)
Language (ref = English)								
Spanish	−1.25 (0.59) *	−1.24 (0.57) *	−0.68 (0.37)	−0.66 (0.34)	−1.26 (1.58)	−1.24 (1.66)	−6.69 (1.76) **	−6.69 (1.77) **
Asian	5.13 (1.22) ***	5.22 (1.29) **	2.55 (0.43) ***	2.66 (0.51) ***	−0.19 (1.46)	−0.05 (1.27)	9.30 (3.35) *	9.29 (3.27) *
Elevated HbEtO levels		−0.62 (0.48)		0.75 (0.25) **		−1.05 (0.91)		0.09 (2.35)
Constant	36.11 (2.38) ***	36.19 (2.37) ***	15.27 (1.29) ***	15.37 (1.24) ***	39.75 (3.35) ***	39.88 (3.36) ***	103.12 (6.02) ***	103.11 (6.03) ***
*R* ^2^	0.24	0.24	0.19	0.21	0.25	0.26	0.44	0.44

* *p* < 0.05, ** *p* < 0.01, *** *p* < 0.001.

**Table 5:** Hierarchical linear regression of predictors of neurocognitive performance and HbEtO levels on combined grip strength.

	Step 1			Step 2		
Variable	*B*	SE	*p*	*B*	SE	*p*
Age (years)	−0.96	0.10	<0.001	−0.96	0.10	<0.001
Serum Cotinine Levels	−0.01	0.01	0.450	−0.00	0.01	0.853
Poverty Ratio	1.06	0.35	0.008	0.98	0.36	0.016
Male Gender (ref = female)	28.69	1.20	<0.001	28.91	1.23	<0.001
Race/Ethnicity (ref = white)						
Hispanic	−7.54	2.35	0.006	−7.27	2.30	0.006
Black	2.01	2.54	0.440	2.42	2.52	0.353
Asian	−6.70	1.77	0.002	−5.94	1.71	0.003
Other	−7.06	8.29	0.408	−6.72	7.58	0.389
Education (ref = college grad)						
<12th Grade	0.07	2.46	0.979	−0.38	2.39	0.876
Highschool	−0.68	1.57	0.670	−0.44	1.60	0.786
Some College	1.28	1.29	0.340	0.81	1.22	0.518
Elevated HbEtO levels				−3.15	1.06	0.009
*R* ^2^		0.69			0.69	

**Table 6:** Independent *t*-tests for background vs. elevated HbEtO levels.

HbEtO Levels	Background		Elevated				
	M	SD	M	SD	*t*	*df*	*p*
CERAD-WL Learning Trials	20.76	0.35	20.01	0.38	1.84	15	0.085
CERAD Delayed Recall	6.74	0.15	5.99	0.25	3.53	15	0.003
Animal Fluency	18.54	0.37	16.72	0.56	2.67	15	0.018
Digit Symbol Substitution Test	52.44	1.30	47.91	1.29	2.24	15	0.041
Combined Grip Strength	65.12	1.43	63.68	2.40	0.60	15	0.559

**Table 7:** Logistic regressions for elevated EtO levels predicting low cognitive scores.

Elevated EtO Levels Predicting Low Scores on Cognitive Tests:	*B*	*SE*	OR	95% CI	*p*
CERAD-WL Trial # 1	0.74	0.26	2.09	[1.20, 3.65]	0.013
CERAD-WL Trial # 3	0.36	0.31	1.43	[0.74, 2.79]	0.270
CERAD Delayed Recall	0.56	0.25	1.76	[1.02, 3.02]	0.042
Animal Fluency	0.58	0.18	1.79	[1.21, 2.64]	0.006
Digit Symbol Substitution Test	0.40	0.28	1.50	[0.82, 2.72]	0.172

CI = confidence interval for odds ratio (OR).

## Data Availability

The data analyzed in this study can be found within the publicly available NHANES database, https://www.cdc.gov/nchs/nhanes/, accessed on 5 June 2024.

## Abbreviations

The following abbreviations are used in this manuscript:

EtO	Ethylene Oxide Gas
CERAD	Consortium to Establish a Registry for Alzheimer’s Disease
NHANES	National Health and Nutrition Examination Survey
DSST	Digit Symbol Substitution test
